# Harnessing Self-Control and AI: Understanding ChatGPT’s Impact on Academic Wellbeing

**DOI:** 10.3390/bs15091181

**Published:** 2025-08-29

**Authors:** Metin Besalti

**Affiliations:** 1 Department of Educational & Psychological Studies, Faculty of Education, University of South Florida, Tampa, FL 33620, USA; metinbesalti@usf.edu; 2 Department of Educational Sciences, Faculty of Education, Artvin Coruh University, 08000 Artvin, Türkiye; besalti@artvin.edu.tr

**Keywords:** ChatGPT usage, self-control, academic wellbeing, generative artificial intelligence, AI in education

## Abstract

The rapid integration of generative AI, particularly ChatGPT, into academic settings has prompted urgent questions regarding its impact on students’ psychological and academic outcomes. Although generative AI holds considerable potential to transform educational practices, its effects on individual traits such as self-control and academic wellbeing remain insufficiently explored. This study addresses this gap through a sequential two-phase design. In the first phase, the ChatGPT Usage Scale was adapted and validated for a Turkish university student population (*N* = 413). Using confirmatory factor analysis and item response theory, the scale was confirmed as a psychometrically valid and reliable one-factor instrument. In the second phase, a separate sample (*N* = 449) was used to examine the relationships between ChatGPT usage, self-control, and academic wellbeing through a mediation model. The findings revealed that higher ChatGPT usage was significantly associated with lower levels of both self-control and academic wellbeing. Additionally, mediation analysis demonstrated that self-control partially mediates the negative relationship between ChatGPT usage and academic wellbeing. The study concludes that while generative AI tools are valuable, their integration into education presents a double-edged sword, highlighting the critical need to foster students’ self-regulatory skills to ensure they can harness these tools responsibly without compromising their academic and psychological health.

## 1. Introduction

The integration of Artificial Intelligence (AI) in education has rapidly evolved, reshaping learning experiences across various academic levels (Zawacki-Richter et al., 2019; Lund & Wang, 2023). Among the various applications of AI, generative AI (GenAI) tools have gained significant attention due to their ability to create content, simulate human-like interactions, and support personalized learning experiences. GenAI refers to systems that leverage advanced natural language processing (NLP) models to generate coherent, contextually relevant, and human-like responses based on input data (Bozkurt, 2023). These tools utilize large-scale pre-trained models, such as the Generative Pre-trained Transformer (GPT), to analyze patterns in language and produce outputs that mimic human communication. ChatGPT, a prime example of GenAI, was launched by OpenAI and rapidly became a transformative tool in educational settings (OpenAI, 2022). It quickly amassed a large user base, reaching an estimated 100 million monthly active users within two months of its release (K. Hu, 2023), making it one of the fastest-growing consumer applications in history. According to a survey from the Digital Education Council (2024), ChatGPT is the most commonly used AI tool among students. Its widespread adoption, user-friendly interface, and significant public attention have positioned it as a dominant and highly accessible model, making it a critical focus for understanding the immediate impacts of generative AI in education (Qian, 2025; Chiu, 2024; Kasneci et al., 2023). It supports a range of academic activities, from writing assistance and coding to problem-solving techniques and data analysis, all delivered through a conversational interface (Farrokhnia et al., 2023).

ChatGPT’s core functionality is based on an architecture that learns and adapts from user interactions. This design allows it to provide personalized support and serve as a virtual tutor, offering real-time feedback, clarifications, and guidance (Shen et al., 2023; Vargas-Murillo et al., 2023). Because of this flexibility, it can address a wide range of questions, such as covering academic research, technical explanations, and creative writing, making it a valuable resource for many learners. A central feature that sets ChatGPT apart from traditional educational technologies is its ability to simulate real-time human interaction, creating a conversational experience that feels natural and engaging (Deng & Lin, 2023). This feature of ChatGPT transforms the learning process for students by enhancing interactivity and personalizing education to meet individual needs.

While ChatGPT offers numerous advantages for learners, it presents a double-edged sword (Shen et al., 2023), raising ethical concerns (Liebrenz et al., 2023), fostering potential dependency (Zhang et al., 2024), and diminishing critical thinking skills (Kasneci et al., 2023). In addition, it may create challenges, including the risk of misuse, over-reliance, and academic dishonesty, highlighting the need for guidance when using it in educational settings (Grassini, 2023; Vargas-Murillo et al., 2023).

Recent reviews have confirmed the growing scholarly interest in the psychological and academic implications of GenAI tools in education. A systematic review by Qian (2025) synthesized findings in higher education settings to evaluate the current use and impact of GenAI in teaching and learning, reporting both positive and negative effects. Pedagogically, GenAI is being deployed for automated feedback and assessment, providing learning support, and, notably, fostering critical skills such as creativity, critical thinking, learning autonomy, and prompt literacy. However, the study also identifies significant challenges, including technical limitations, quality and ethical concerns (e.g., misinformation, bias, plagiarism), pedagogical issues (e.g., AI feedback lacking depth, assessment validity), and a crucial concern regarding student AI literacy and dependency, where students may outsource cognitive effort and diminish their independent problem-solving abilities.

Mustafa et al. (2024) echoed these issues in a meta-synthesis. These include ethical concerns (e.g., data privacy, bias), technical limitations (e.g., cost, lack of human elements), and rising concerns over student AI literacy and dependency, particularly the tendency to over-rely on AI tools at the expense of independent thinking and academic integrity. Similarly, Zhai et al. (2024) conducted a systematic review to examine the cognitive consequences of students’ over-reliance on GenAI tools. Their findings reveal that although these tools enhance efficiency, excessive dependence can diminish critical thinking, analytical reasoning, and the ability to make independent decisions. These findings highlight the importance of promoting AI literacy and fostering pedagogical approaches that encourage active, critical engagement with GenAI. To maximize ChatGPT’s positive impact, it is crucial to strike a balance between leveraging its advantages and addressing its limitations, ensuring that the development of students’ core cognitive and ethical competencies remains uncompromised.

### 1.1. ChatGPT and Self-Control

While the transformative potential of ChatGPT is undeniable, its integration into educational settings raises important questions about the role of individual psychological traits, such as self-control, in moderating the usage and impact of ChatGPT (Rodríguez-Ruiz et al., 2025). Self-control is the process by which individuals consciously decide to take responsibility for their own actions (Rosenbaum, 1993). Self-control in academic contexts is particularly important because it enables students to prioritize tasks, maintain focus, and continue learning even when distractions abound (Duckworth et al., 2019). However, the introduction of highly accessible GenAI tools like ChatGPT presents both opportunities and challenges for self-control.

On the one hand, ChatGPT can provide students with immediate support and solutions (Ngo, 2023), easing cognitive load (Patac & Patac, 2025) and creating an interactive and dynamic learning environment (Sandu et al., 2024). For individuals with strong self-control, these tools may serve as valuable assets to enhance efficiency and deepen learning. On the other hand, students with lower levels of self-control may face unique challenges when using ChatGPT. The tool’s ease of access and capacity to generate instant responses may encourage users to avoid critical thinking and problem-solving processes, increasing dependence and reducing intrinsic motivation (Rodríguez-Ruiz et al., 2024; Vargas-Murillo et al., 2023). This dynamic has been linked to the concept of AI dependency, wherein over-reliance on GenAI tools reduces students’ ability to self-regulate and independently overcome academic challenges (Zhang et al., 2024).

AI dependency is defined as an excessive reliance on AI technologies not only in academic tasks but also in everyday life and social interactions (Zhang et al., 2024). This dependency involves both the frequent use of AI tools and a psychological reliance on these technologies (Morales-García et al., 2024). Zhang et al. (2024) found that academic stress and performance expectations mediate the relationship between academic self-efficacy and AI dependency among university students. This suggests that students with lower academic self-efficacy may experience higher levels of academic stress and have higher performance expectations, which in turn may lead to increased reliance on AI tools.

Research highlights the interplay between self-control and ChatGPT usage. For example, the research conducted by Prayoga and Wakhid (2024) revealed that individuals with higher levels of self-control exhibited a significantly reduced tendency to develop an over-reliance on AI chatbots. Their findings suggest that self-control plays a critical role in moderating the extent to which users depend excessively on AI-driven conversational tools, highlighting the importance of self-regulatory mechanisms in fostering balanced and mindful engagement with such technologies. Similarly, Rodríguez-Ruiz et al. (2025) investigated the relationship between psychopathy traits and the use of AI tools among university students, specifically examining the mediating effect of self-control. They found that low self-control plays a role in the frequency of AI tool use among university students and mediates the relationship between psychopathy traits and the frequency of AI tool use. Additionally, low self-control was negatively associated with using AI tools to solve everyday doubts.

In a similar study, Rodríguez-Ruiz et al. (2024) explored the relationship between the use of AI tools, self-control, self-esteem, and self-efficacy among university students. The study found that low self-control is a significant predictor of a higher frequency of AI tool use among university students. Moreover, low self-control is associated with specific types of AI tools use, namely increased use for social interaction and academic task completion and decreased use for solving everyday doubts. Another study done by Feng et al. (2023) investigated the mediating role of self-control in the association between ChatGPT usage and online learning burnout among Chinese college students. The study found a significant negative association between active ChatGPT usage and college students’ self-control, which indicates that a more frequent use of ChatGPT is associated with a lower level of self-control. They also found that the increased use of ChatGPT contributes to a decrease in self-control, which, in turn, leads to higher levels of online learning burnout.

In summary, research highlights a complex relationship between the use of AI tools and self-control. The increased use of AI tools, such as ChatGPT, is generally associated with lower levels of self-control. The ease of access to immediate answers and solutions offered by ChatGPT may discourage sustained effort and attention, thereby potentially hindering the development and maintenance of self-control. Furthermore, individuals with lower self-control tend to use AI tools more frequently for specific purposes, such as completing academic work. The impact of self-control goes beyond the individual level, shaping broader patterns of adoption and integration of AI tools within educational environments.

### 1.2. ChatGPT Usage and Academic Wellbeing

The increasing integration of AI tools, such as ChatGPT, into higher education is significantly influencing students’ academic and personal lives, notably their academic wellbeing (Klimova & Pikhart, 2025). Academic wellbeing is a key measure of educational success because it affects students’ motivation, engagement, and ability to handle challenges (Bird & Markle, 2012; Arslan et al., 2022). As AI tools become embedded in learning routines, they offer students new ways to access information, clarify difficult concepts, and receive instant feedback, which can enhance students’ sense of competence and reduce academic stress (Shahzad et al., 2024). These tools can also boost students’ learning acquisition by providing personalized feedback, step-by-step problem-solving techniques, and clear explanations on complex topics (Jo, 2024; Labadze et al., 2023). However, the relationship between ChatGPT usage and students’ academic wellbeing is multifaceted and reveals both positive and negative effects (Klimova & Pikhart, 2025).

Several studies suggest positive correlations between the use of AI tools and academic wellbeing. For example, Ajlouni et al. (2024) investigated the relationship between the intensity of the academic use of AI chatbots and academic wellbeing among undergraduate students at the University of Jordan. They found a significant positive correlation between the intensity of the academic use of AI chatbots and academic wellbeing among undergraduate students. Similarly, Shahzad et al. (2024) examined the impact of ChatGPT usage and social media use on the academic performance and psychological wellbeing of Chinese university students. They found that the use of ChatGPT had a positive impact on students’ academic performance and overall wellbeing. In line with this study, Rezai et al. (2024) investigated the correlation between the use of ChatGPT and wellbeing among English as a Foreign Language (EFL) learners in Iran, with a specific focus on the mediating role of emotion regulation. The results revealed a significant positive correlation between the use of ChatGPT and wellbeing among Iranian EFL learners.

However, the use of GenAI tools in educational settings also presents challenges and negative impacts on academic wellbeing. Excessive reliance on GenAI tools can undermine critical thinking, diminish feelings of achievement, and increase dependence, thus removing the personal reward of academic success (Zhang et al., 2024; Duong et al., 2025). This issue becomes more serious when students skip essential learning steps such as problem-solving techniques, analysis, and reflection, in favor of AI-generated shortcuts. Moreover, the compulsive use of ChatGPT, which may result from limited self-regulation and self-control, can lead to technology-related stress and a feeling of isolation, both of which harm mental and academic wellbeing (Duong et al., 2025).

### 1.3. Research Aims and Hypotheses

Despite increasing research on the use of ChatGPT in education, its broader effects remain underexplored. Previous studies have highlighted benefits such as improved academic efficiency, personalized learning support, and relief from the stress associated with complex tasks (Ajlouni et al., 2024; Rezai et al., 2024). However, much of this work has concentrated on broad outcomes and has not thoroughly examined how ChatGPT usage interacts with students’ psychological traits, particularly self-control, in shaping their academic wellbeing. Self-control has been consistently linked to both technology use behaviors (e.g., Burnell et al., 2023) and academic wellbeing (e.g., Hofmann et al., 2014). Specifically, prior studies have shown that individuals with lower self-control tend to engage more frequently in potentially distracting or passive forms of technology use, which can in turn impact their academic wellbeing (Troll et al., 2021; Mei et al., 2016). Although some research has investigated self-regulation as a predictor of ChatGPT usage (Zhang et al., 2024), no studies have explored whether students’ self-control mediates the relationship between ChatGPT usage and academic wellbeing. By introducing self-control as a mediator, the study aims to explore whether the effect of ChatGPT usage on academic wellbeing operates through a behavioral regulatory mechanism.

To address this gap, the current study adapts the ChatGPT Usage Scale developed by Abbas et al. (2024) for a Turkish context and then examines ChatGPT’s relationships with self-control and academic wellbeing among Turkish university students. While the original scale focused on the psychometric measurement of ChatGPT usage, validating and testing its reliability in the Turkish population will ensure its suitability for cross-cultural research. Beyond this psychometric contribution, the mediating role of self-control in the relationship between ChatGPT usage and academic wellbeing is investigated. By doing so, it is aimed to provide deeper insights into the multifaceted ways in which ChatGPT usage interacts with psychological and academic variables, thereby enriching the literature on ChatGPT’s impact in educational settings. In this context, the following hypotheses will be answered in the study:

**H1.**  *The ChatGPT Usage Scale is psychometrically valid and reliable.*

**H2.**  *There is a significant negative relationship between ChatGPT usage and self-control.*

**H3.**  *There is a significant negative relationship between ChatGPT usage and academic wellbeing.*

**H4.**  *Self-control has a mediating role in the relationship between ChatGPT usage and academic wellbeing.*

## 2. Methods

The research was conducted in two sequential phases to ensure both the validation of the measurement tool and the examination of the hypothesized relationships. The first phase of the study was aimed at adapting the ChatGPT Usage Scale to the Turkish language and examining its psychometric properties. The scale was developed by Abbas et al. (2024), and three experts fluent in both English and Turkish translated the scale into Turkish. To ensure accuracy, a back-translation process was carried out and necessary corrections were made to the final version of the scale. The scale was administered online to a Turkish sample, and data were subsequently collected using Google Forms. In the initial phase of the study, confirmatory factor analysis (CFA) was performed on the ChatGPT Usage Scale. Additionally, the scale was evaluated using item-total correlations and item response theory (IRT). Reliability analyses were also included in this phase.

Following the psychometric evaluation of the ChatGPT Usage Scale, the second phase of the study aimed to examine the associations among ChatGPT usage, self-control, and academic wellbeing, while also testing these variables within a hypothesized model. At this phase, the initial focus was to investigate the interrelationships among these variables.

### 2.1. Participants and Procedure

In both phases of the study, participants were recruited through convenience sampling based on the following inclusion criteria: (1) enrollment as a university student, (2) prior experience using ChatGPT, and (3) voluntary consent to participate in the study. Between September and December 2024, a total of 413 participants were recruited for the first phase of the study. Among them, 32.2% were male (*n* = 133) and 67.8% were female (*n* = 280), with a mean age of 21.55 years (*SD* = 2.04) and an age range from 17 to 29 years. Educational background showed that most of the participants were at the undergraduate level at 94.9% (*n* = 392), while 5.1% (*n* = 21) were at the postgraduate level. Regarding socio-economic status, 28.8% (*n* = 119) were classified as having low status, 67.3% (*n* = 278) medium status, and 3.9% (*n* = 16) high status.

For the second phase, a total of 449 participants were recruited between January and February 2025. Among them, 58.8% were female (*n* = 264) and 41.2% were male (*n* = 185), with a mean age of 21.83 years (*SD* = 3.07) and an age range from 17 to 37 years. Educational background showed that most of the participants were at the undergraduate level at 90.6% (*n* = 407), while 9.4% (*n* = 42) were at the postgraduate level. Regarding socio-economic status, 45.4% (*n* = 204) were classified as having low status, 41.9% (*n* = 188) medium status, and 12.7% (*n* = 57) high status. Detailed demographic characteristics of the participants for both phases of the study are provided in Table 1.

The online form link was primarily distributed through university-related channels on social media, inviting participants to complete the research scales. To mitigate the risk of responses from individuals outside the intended population, inclusion criteria were clearly stated at the beginning of the survey, and participants were required to confirm they were current university students. Informed consent was obtained, participation was voluntary, and no compensation was offered. Participants could withdraw at any time, and the form could only be submitted if all questions were answered. The questionnaire consisted of three sections. The first section included a filter question to identify participants who had used GenAI tools, such as ChatGPT (e.g., “*Have you used any generative AI tools in your studies? Yes/No*”). Those who answered “No” were not allowed to proceed. The second section collected demographic information (gender, age, socio-economic status, and education), while the final section contained the ChatGPT Usage Scale along with additional validated measures. To ensure data quality, several precautions were taken: attention-check items were included (e.g., “*Select ‘I agree’ for this question*”), response times were monitored to exclude overly quick completions, and duplicate entries were filtered out. Participants were also reminded of the importance of honest and thoughtful responses before beginning.

### 2.2. Measures

#### 2.2.1. ChatGPT Usage Scale

The ChatGPT Usage Scale, developed by Abbas et al. (2024) to assess the overall usage of ChatGPT, was adapted into Turkish for the current study. The scale consisted of 8 positively worded items (e.g., “*I use ChatGPT for my course assignments*”) rated on a 6-point Likert scale ranging from 1 (*never*) to 6 (*always*). The original study reported a Cronbach’s alpha of α = 0.91, while the current study found an internal consistency of α = 0.94.

#### 2.2.2. Brief Self-Control Scale

The 13-item inventory was developed by Tangney et al. (2004), and the Turkish adaptation was conducted by Nebioglu et al. (2012). Participants responded on a 5-point Likert scale (1 = *not at all true of me* to 5 = *totally true of me*). The scale included two dimensions: impulsivity and self-discipline. Reported Cronbach’s alpha values for the subscales were 0.87 and 0.81, respectively, with an overall scale reliability of 0.83.

#### 2.2.3. Subjective Academic Wellbeing Scale

The scale developed by Arslan et al. (2022) consisted of a total of 7 items. The scale had a one-dimensional structure and a 5-point Likert-type rating (1 = *Never*, 5 = *Always*). Possible scores from the scale ranged from 7 to 35, and increasing scores indicated a higher subjective academic wellbeing. The Cronbach’s alpha reliability coefficient for the scale was reported to be α = 0.95.

### 2.3. Translation Process

The ChatGPT Usage scale was translated from English to Turkish following a rigorous, multi-step process. This approach, aligned with international standards (International Test Commission, 2017), ensured both linguistic accuracy and cultural relevance. The translation process was conducted after obtaining formal permission from the original scale’s co-author. The translation–back-translation methodology was employed to ensure linguistic and cultural accuracy. The scale items were first translated into Turkish by three experts with proficiency in both educational technology and English. These individual translations were then compared and synthesized into a single, unified Turkish version. This version was further refined by a panel of three experts with doctoral degrees in educational technology, measurement and evaluation, and the Turkish language. To verify the quality of the translation, the final Turkish version was back-translated into English by an independent expert. This back-translated text was then compared against the original English scale to check for semantic integrity. Any inconsistencies identified were addressed, and the Turkish version was revised accordingly. Finally, the Turkish scale was pilot tested with a sample of 15 adults in order to confirm its clarity and comprehensibility. Based on the feedback received, final corrections were made before the scale was used for the main study (see Supplementary Materials for details).

### 2.4. Data Analysis

In the first phase of the study, CFA was used to evaluate the 8-item form of the scale using maximum likelihood estimation in AMOS Graphics. The Comparative Fit Index (CFI), Normed Fit Index (NFI), Incremental Fit Index (IFI), Tucker–Lewis Index (TLI), and Standardized Root Mean Square Residual (SRMR) were used to evaluate the model fit. Item-total correlations of the scale and reliability analysis of other scales were also analyzed. The validation process involved two main steps: (i) examining descriptive statistics for each item, including means and standard deviations, and (ii) evaluating internal consistency reliability through Cronbach’s alpha, McDonald’s omega, and composite reliability (CR), with a threshold of 0.70 indicating satisfactory reliability (McDonald, 1999). Since the original scale developed by Abbas et al. (2024) has an established theoretical structure and factorial validity, CFA was conducted to verify the scale’s structure in the Turkish context. Model adequacy was judged based on established fit indices: NNFI (non-normed fit index) values above 0.90 (preferably ≥ 0.95), CFI (comparative fit index) values above 0.90 (preferably ≥ 0.95), RMSEA (root mean square error of approximation) values below 0.10 (ideally ≤ 0.08), and SRMR (standardized root mean square residual) values below 0.08 (ideally ≤ 0.06) (Fornell & Larcker, 1981; L. T. Hu & Bentler, 1999; Kline, 2011).

In the second phase of the study, initial descriptive analyses of the study variables were conducted using the SPSS statistical software v.27 (IBM Corp., 2020). Following this, correlation analysis was employed to assess the relationships among ChatGPT usage, self-control, and academic wellbeing. Once these relationships were established, structural equation modeling (SEM) was utilized to evaluate the proposed theoretical model, with the analyses being performed using the AMOS software. Covariance-based SEM was conducted using AMOS Graphics, as this approach is well suited for theory testing and validation when the model is pre-specified and the sample size is sufficient (Awang et al., 2015). SEM was selected for its ability to simultaneously test the relationships among latent variables and their indicators, providing a comprehensive evaluation of the model fit and construct validity. To address differences in response scales (i.e., 5-point vs. 6-point Likert), all variables included in SEM were z-standardized before analysis to ensure comparability and avoid distortion. This approach was chosen because it offers a robust estimation of indirect effects using bias-corrected bootstrapping, which enhances statistical power and does not rely on assumptions of normality in the sampling distribution (Hayes, 2018).

For mediation analysis, the process began with an assessment of the measurement model, followed by an evaluation of the structural model, as recommended by Anderson and Gerbing (1988). The model fit was assessed based on established criteria, with threshold values for the fit indices (CFI, GFI, TLI, and IFI > 0.90; SRMR and RMSEA < 0.08) guided by Hoyle and Panter (1995).

Data analyses were performed using SPSS Statistics v.27 (IBM Corp., 2020) and Jamovi (Jamovi Project, 2023) for descriptive statistics and reliability analyses. CFA and correlation analyses were conducted using JASP Team (2024) version 0.19. Additionally, the R Core Team (2021) software was utilized to assess discriminant and convergent validity. Mediation analysis was carried out using the Hayes PROCESS macro (Hayes, 2018), which allows for robust testing of indirect effects via bootstrapping.

## 3. Results

### 3.1. Results for Phase 1

During the first phase of the study, CFA was conducted to evaluate the 8-item form of the ChatGPT Usage Scale. The fit indices for this form were then examined. The CFA results indicated an acceptable model fit for the 8-item ChatGPT Usage Scale: χ^2^ (215.312, N = 413) = 10.766, *p* < 0.001; CFI = 0.935; NFI = 0.929; IFI = 0.935; TLI = 0.909; SRMR = 0.044. Thus, the 8-item structure of the ChatGPT Usage Scale was validated. Additionally, convergent validity for the eight items was assessed following CFA. The Average Variance Extracted (AVE) was calculated as 0.73, exceeding the recommended threshold of 0.50 (Bagozzi & Yi, 1988), indicating satisfactory convergent validity. Factor scores, descriptive statistics, and item-total correlations are shown in Table 2.

#### 3.1.1. Measurement Invariance

After CFA validated the factor structures of the ChatGPT Usage Scale, an assessment was conducted to check if the scale measures distinct constructs consistently across groups. Specifically, CFA was applied separately to data sets split by gender, with results displayed in Table 3.

#### 3.1.2. Item Response Theory

Item response theory (IRT) is widely acknowledged as an essential methodological framework for addressing a range of measurement challenges (Harwell, 1997). Distinct from summative scoring approaches, IRT prioritizes the independent evaluation of individual items (Baker & Kim, 2017). Given the Likert-scale structure of the ChatGPT Usage Scale, IRT analysis is expected to yield a more nuanced understanding of response patterns. Baker (2001, p. 34) posits that an α value exceeding 1 denotes high item discrimination. In the conducted IRT analysis, all items demonstrated an α value greater than 1, with results reported in Table 4.

#### 3.1.3. Reliability Analysis

Reliability analyses for the 8-item ChatGPT Usage Scale were performed employing Cronbach’s Alpha, McDonald’s Omega, and Guttman’s Lambda. The findings, detailed in Table 5, confirm that the scale constitutes a reliable instrument for measurement.

### 3.2. Results for Phase 2

In the second phase, the study variables were first examined through descriptive analyses, followed by SEM to test the proposed theoretical model. The results revealed that ChatGPT usage demonstrated a significant, very weak negative association with self-control (β = −0.13, *p* < 0.05), suggesting that an increased use of ChatGPT is linked to slightly lower levels of self-regulatory ability. In turn, self-control showed a significant, weak positive association with subjective academic wellbeing (β = 0.21, *p* < 0.01), indicating that greater self-control corresponds with higher perceived subjective academic wellbeing. ChatGPT usage also had a significant, very weak negative total effect on subjective academic wellbeing (*c* = −0.10, *p* < 0.05).

**Figure 1:**
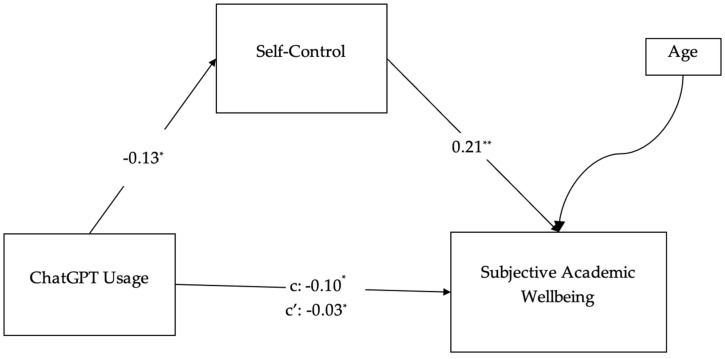
Hayes’ process modeling for the simple mediation model. Notes: * *p* < 0.05. ** *p* < 0.01. Age: Control variable.

A mediation model was tested using the adapted scale, employing Hayes’ PROCESS macro for mediation analysis (Hayes, 2018). The model aimed to examine the mediating role of self-control in the relationship between ChatGPT usage and subjective academic wellbeing. In the model, age was included as a control variable to account for potential variance associated with participants’ demographic characteristics. Although not the primary focus of the analysis, controlling for age allowed for a more accurate estimation of the relationships between ChatGPT usage, self-control, and academic wellbeing by minimizing potential confounding effects related to age-related differences in digital tool usage or self-regulatory behavior. The analysis revealed a partial mediating effect of self-control in this relationship. Specifically, ChatGPT usage was found to negatively predict self-control (*β* = −0.13, *p* < 0.05), self-control positively predicted subjective academic wellbeing (*β* = 0.21, *p* < 0.001), and ChatGPT usage also directly and negatively predicted subjective academic wellbeing (*β* = −0.10, *p* < 0.05). These findings suggest that lower self-control partially explains the negative relationship between ChatGPT usage and students’ academic wellbeing. The findings are visually represented in Figure 1.

#### Bootstrapping

To assess the significance of the results obtained from the Hayes PROCESS mediation analysis, bootstrap analysis with 5000 resamples and a 95% confidence interval was conducted. The findings indicated that both the direct effect (*β* = −0.05, *p* < 0.001, 95% CI = −0.11 to −0.01) and the indirect effect of self-control as a mediator between ChatGPT usage and subjective academic wellbeing (*β* = −0.03, *p* < 0.001, 95% CI = −0.07 to −0.01) were statistically significant, as both confidence intervals excluded zero. Table 6 presents the results of bootstrap analysis of the mediation effect.

## 4. Discussion

The proliferation of GenAI in education has not only transformed how students access information and engage with course materials but also raised pressing questions about its broader psychological and academic implications. While much of the emerging literature has focused on ChatGPT’s capacity to generate text, foster creativity, and provide individualized feedback, less is known about how the regular use of such tools intersects with students’ self-control and overall academic wellbeing. In the present study, the ChatGPT Usage Scale was first adapted and validated for the Turkish population, and its factor structure and reliability were confirmed. Relationships between ChatGPT usage, self-control, and academic wellbeing were then explored. Finally, mediation analysis was conducted to determine whether self-control explains the relationship between ChatGPT usage and academic wellbeing.

The findings obtained within the first phase of the study are that the ChatGPT Usage Scale is both a valid and a reliable measurement instrument. According to CFA, the one-factor structure of the ChatGPT Usage Scale was supported in its Turkish adaptation, in line with the original validation conducted on an English-speaking sample in Pakistan (Abbas et al., 2024). All fit-index values fell within the ranges recommended in the literature (Hoyle & Panter, 1995), and item factor loadings were observed at acceptable levels (Comrey & Lee, 2013). In addition, gender-based measurement invariance testing demonstrated a statistically equivalent model fit for both male and female participants, indicating that the ChatGPT Usage Scale functions comparably across genders within the Turkish context. Finally, item response theory analyses revealed that each scale item achieved very high classification levels according to Baker’s criteria (Baker, 2001). Discriminatory power, defined as the probability of distinguishing between two randomly selected respondents, was also high for every item (Smalldon & Moffat, 1973), confirming that response patterns on all items were distinctive.

Furthermore, the reliability of the ChatGPT Usage Scale was assessed through multiple metrics. Values for Cronbach’s alpha, McDonald’s omega, Guttman’s lambda, and composite reliability exceeded the thresholds recommended in the literature (Shrout & Fleiss, 1979), indicating that the scale is a robust measurement tool. These results confirm the scale’s one-factor structure, establish measurement invariance, demonstrate strong item discrimination, and indicate reliability levels within accepted psychometric standards. In addition, the reliability of the ChatGPT Usage Scale was evaluated using several statistical measures. The results for Cronbach’s alpha, McDonald’s omega, Guttman’s lambda, and composite reliability all surpassed established benchmarks (Shrout & Fleiss, 1979), supporting the scale’s robustness as a measurement instrument. Additionally, the scale’s ability to produce reliable results with large sample groups demonstrates its effectiveness for wide-scale research applications.

In the second phase, a significant negative relationship was found between ChatGPT usage and self-control, as well as between ChatGPT usage and academic wellbeing. Moreover, self-control was shown to partially mediate the relationship between ChatGPT usage and academic wellbeing. These findings suggest that increased reliance on ChatGPT is associated with a reduced capacity for self-regulation, which in turn contributes to a decline in students’ academic wellbeing. This negative association between ChatGPT usage and self-control aligns with previous studies that raise concerns about the cognitive and behavioral implications of frequent AI usage. For instance, Rodríguez-Ruiz et al. (2024, 2025) and Feng et al. (2023) noted that individuals with lower self-control are more likely to turn to AI tools for immediate assistance, particularly in academic settings, reinforcing a dependency loop. This finding is particularly relevant in the context of higher education, where students may face increasing pressure to perform efficiently and may thus be more inclined to outsource cognitive effort to AI tools.

Likewise, the finding that higher ChatGPT usage is associated with lower academic wellbeing supports existing concerns that overuse of GenAI can undermine students’ sense of achievement (Duong et al., 2025; Zhang et al., 2024). Although GenAI may temporarily reduce stress by offering quick academic support, the present study suggests that this effect is outweighed by the long-term psychological costs. In particular, students may miss out on the development of deeper cognitive skills and personal mastery, both of which are critical for sustaining academic wellbeing (Van Tonder et al., 2022). This interpretation resonates with the literature on technology-related stress, which points to compulsive tool use and reduced personal agency as factors that erode academic wellbeing (Duong et al., 2025; Whelan et al., 2022).

The identification of self-control as a partial mediator is a key contribution of this study. It indicates that while ChatGPT usage directly and negatively affects academic wellbeing, part of this impact operates through its negative association with self-control. This suggests that students with lower self-control may be more likely to engage with ChatGPT in ways that undermine meaningful academic engagement, such as relying on it for shortcuts rather than as a learning aid. These findings underscore the importance of cultivating students’ self-regulatory capacities, particularly in the context of GenAI integration into learning environments. Becoming self-directed in reflecting on and adapting one’s cognitive, motivational, and emotional efforts during learning is known to support both academic and personal wellbeing (Villavicencio & Bernardo, 2013) and may act as a protective factor against the negative effects of over-reliance on AI tools. Within this framework, promoting AI literacy alone is insufficient. Educators must also help students develop the self-regulatory skills needed to engage with such tools critically and purposefully.

These results were expected within the context of this study, as the university students experienced increased exposure to GenAI tools without substantial guidance on their ethical and pedagogical use. As institutions race to adopt AI, a gap persists in students’ AI literacy and self-regulation strategies, which may explain why overuse is linked to negative outcomes. This underscores the need for higher education institutions to support students in developing critical, balanced, and reflective approaches to GenAI tool use.

### 4.1. Limitations

Although this study offers valuable insights, it is not without limitations. First, the use of a cross-sectional design constrains the capacity to establish causal relationships. Although the mediation model is grounded in theory, longitudinal studies are needed to more definitively establish the directional relationships between ChatGPT usage, self-control, and academic wellbeing over time. Second, relying solely on self-reported data may introduce common method bias as well as social desirability bias. Future research could benefit from incorporating objective measures of ChatGPT usage and behavioral assessments of self-control and academic wellbeing. Third, the sample consisted primarily of Turkish university students, which may limit the generalizability of the findings to other cultural contexts, educational levels, or age groups. Additionally, the appropriateness of the convenience sampling method and the voluntary participation approach may limit the representativeness of the sample, and thus, caution is warranted in generalizing the results.

### 4.2. Implications

Despite its limitations, the study offers several meaningful implications for educational practice, policy, and future research. First, the study offers a scientific contribution through the adaptation and validation of the ChatGPT Usage Scale for Turkish university students. This validated instrument provides researchers with a reliable tool to measure ChatGPT usage in educational settings. Additionally, it contributes to the literature by being the first to empirically test and confirm the mediating role of self-control in the relationship between ChatGPT usage and academic wellbeing, addressing a notable gap in the research. The findings highlight the importance of fostering self-control among students in AI-based learning environments. Educators and institutions are encouraged to implement strategies and programs that support the development of students’ self-regulation skills, particularly in the context of GenAI-supported learning tools.

Practically, the findings underscore the importance of fostering self-control skills in students as GenAI tools become more integrated into educational settings. Educators and institutions should not simply ban or permit these tools but rather develop pedagogical strategies that promote mindful and balanced engagement to promote academic wellbeing. This could include training students on how to use GenAI tools as a supplement for learning rather than a replacement for critical thinking. In addition, early identification and intervention mechanisms can be designed to detect patterns of excessive GenAI usage and its potential impact on academic wellbeing. Integrating digital literacy and self-regulation into the curriculum can help students develop healthier habits with AI tools. This includes training students not only in technical usage but also in the ethical, cognitive, and motivational implications of their interactions with GenAI. Furthermore, the findings support rethinking traditional assessment models. As AI usage increases, educators may need to revise evaluation strategies to better reflect authentic, independent learning. Support for educator training in AI integration, digital wellbeing, and pedagogical innovation is also vital to ensure responsible and effective adoption of emerging technologies. Researchers may also consider developing interventions aimed at strengthening students’ self-regulatory capacities to mitigate the potential negative impacts of AI dependency on their academic and psychological wellbeing.

## 5. Conclusions

This research embarked on a two-phase investigation into the role of ChatGPT in the academic lives of university students. During the first phase, the ChatGPT Usage Scale was successfully adapted and validated for a Turkish-speaking population, demonstrating robust psychometric properties, including a stable one-factor structure, measurement invariance across genders, and high reliability. In the second phase, this validated scale was employed to investigate the relationships between ChatGPT usage, self-control, and academic wellbeing. The findings revealed a negative association between higher ChatGPT usage and both self-control and academic wellbeing. Critically, the study established that self-control acts as a partial mediator, suggesting that the detrimental effect of ChatGPT usage on academic wellbeing is partly explained by a corresponding decrease in students’ self-control. This highlights the pivotal role of individual psychological characteristics in shaping the outcomes of student interaction with AI technologies.

In conclusion, while GenAI tools like ChatGPT offer undeniable potential to support learning, their integration into education presents a “double-edged sword” (Shen et al., 2023). The findings of this study argue for a cautious and informed approach, emphasizing that the benefits of AI cannot be realized without considering and actively supporting the development of students’ core cognitive and self-regulatory competencies. The key takeaway is that fostering self-control is essential for ensuring that students can harness the power of AI responsibly and maintain their academic wellbeing in an increasingly digital educational landscape.

## Figures and Tables

**Table 1:** Demographic information of participants.

Variable	First Phase Frequency (%)	Second Phase Frequency (%)
*Gender*		
Male	133 (32.2)	185 (41.2)
Female	280 (67.8)	264 (58.8)
*Education Level*		
College student	392 (94.9)	407 (90.6)
Graduate student	21 (5.1)	42 (9.4)
*Socio-Economic Situation*		
Low	119 (28.8)	204 (45.4)
Middle	278 (67.3)	188 (41.9)
High	16 (3.9)	57 (12.7)
*Frequency of Using Generative AI Tools*		
Always	134 (32.4)	121 (27.0)
Very often	173 (41.9)	182 (40.5)
Sometimes	96 (23.2)	120 (26.7)
Rarely	10 (2.5)	26 (5.8)

**Table 2:** Factor loading, descriptive statistics, and item-total correlations.

Item	Factor Loading	Mean	*SD*	Item-Total Correlations
ChatGPT-1	0.88	2.91	1.53	0.83
ChatGPT-2	0.89	2.87	1.52	0.85
ChatGPT-3	0.91	2.95	1.53	0.88
ChatGPT-4	0.83	2.18	1.41	0.78
ChatGPT-5	0.82	2.68	1.41	0.77
ChatGPT-6	0.81	2.34	1.41	0.75
ChatGPT-7	0.83	2.80	1.49	0.78
ChatGPT-8	0.88	2.41	1.46	0.84

**Table 3:** Fit indices of gender invariance.

Invariance	χ^2^	*df*	IFI	CFI	SRMR	∆CFI	∆RMSEA
Males	119.102	20	0.90	0.90	0.058	–	–
Females	118.639	20	0.95	0.95	0.041	–	–
Configural invariance	237.741	40	0.93	0.93	0.041	–	–
Metric invariance	263.909	47	0.93	0.93	0.038	0.004	0.03
Scalar invariance	274.970	54	0.93	0.93	0.037	0.006	0.01

**Table 4:** IRT results for the 8-item ChatGPT Usage Scale.

Item	*a* Coefficient	SE	Confidence Interval	*z*	*p* > |*z*|
Item 1	4.58	0.38	3.38–5.32	12.07	0.001
Item 2	5.14	0.44	4.28–6.00	11.69	0.001
Item 3	6.21	0.58	5.07–7.36	10.63	0.001
Item 4	3.31	0.29	2.75–3.87	11.58	0.001
Item 5	2.71	0.22	2.29–3.14	12.42	0.001
Item 6	2.64	0.22	2.21–3.08	11.86	0.001
Item 7	2.86	0.23	2.42–3.31	12.59	0.001
Item 8	3.94	0.33	3.28–4.59	11.75	0.001

**Table 5:** Reliability analysis of the 8-item ChatGPT Usage Scale.

Analysis	(*N* = 413)
Cronbach’s alpha	0.948
McDonald’s omega	0.948
Gutmann’s lambda	0.949

**Table 6:** Bootstrap analysis of direct and indirect effects (*N* = 449).

Effect	*β*	SE	95% CI	
			Lower	Upper
Direct Effect: ChatGPT Usage → Academic Wellbeing	−0.05	0.026	−0.11	−0.01
Indirect Effect: ChatGPT Usage → Self-Control → Wellbeing	−0.03	0.015	−0.07	−0.01
Total Effect	−0.08	0.36	−0.14	−0.02

Notes: *β*: Unstandardized path coefficient. CI: Confidence intervals. SE: Standard error.

## Data Availability

The data presented in this study are available upon request from the corresponding author due to restrictions related to participant privacy concerns.

## Supplementary Materials

The following supporting information can be downloaded at: https://www.mdpi.com/article/10.3390/bs15091181/s1; Table S1: English and Turkish versions of the ChatGPT Usage Scale.

## Abbreviations

The following abbreviations are used in this manuscript:

AI	Artificial Intelligence
GenAI	Generative Artificial Intelligence
EFL	English as a Foreign Language
IRT	Item Response Theory
SEM	Structural Equation Modeling (SEM)
CFA	Confirmatory Factor Analysis
CFI	Comparative Fit Index
NFI	Normed Fit Index
IFI	Incremental Fit Index
TLI	Tucker–Lewis Index
SRMR	Standardized Root Mean Square Residual
RMSEA	Root Mean Square Error of Approximation
