# Supplementary material for: Harnessing Self-Control and AI: Understanding ChatGPT’s Impact on Academic Wellbeing
Source: Behav Sci (Basel). 2025 Aug 29;15(9):1181. doi: 10.3390/bs15091181 (PMC12466394; doi:10.3390/bs15091181)
Supplement: Supplementary file 1 [file behavsci-15-01181-s001.zip › behavsci-3752696-supplementary.pdf]

Supplementary Materials

Table S1: English and Turkish versions of the ChatGPT Usage Scale

	English version	Turkish version
	Instructions: Please indicate how often you engage in the following activities using ChatGPT. Use the scale below to respond:<br>1 = Never 2 3 4 5 6 = Always	Yönerge: Lütfen aşağıdaki aktiviteleri ChatGPT kullanarak ne sıklıkta gerçekleştirdiğinizi belirtiniz. Cevaplarınızı aşağıdaki ölçeği kullanarak veriniz:<br>1 = Asla 2 3 4 5 6 = Her Zaman
1.	I use ChatGPT for my course assignments.	ChatGPT'yi ödevlerimi yapmak için kullanırım.
2.	I use ChatGPT for my academic activities.	ChatGPT'yi akademik faaliyetlerim için kullanırım.
3.	I use ChatGPT for my course projects.	ChatGPT'yi ders projelerimde kullanırım.
4.	I am addicted to ChatGPT when it comes to studies.	Çalışmalar söz konusu olduğunda, ChatGPT'ye bağımlıyım.
5.	I rely on ChatGPT for my studies.	Çalışmalarım için ChatGPT'ye güvenirim.
6.	I use ChatGPT to prepare for my tests or quizzes.	ChatGPT'yi sınav veya testlere hazırlanmak için kullanırım.
7.	I use ChatGPT to learn course-related concepts.	ChatGPT'yi dersle ilgili kavramları öğrenmek için kullanırım.
8.	ChatGPT is part of my campus life.	ChatGPT, okul hayatımın bir parçasıdır.

Note: English version of the items can be found in Abbas et al. (2024).

References

Abbas, M., Jam, F. A., & Khan, T. I. (2024). Is it harmful or helpful? Examining the causes and consequences of generative AI usage among university students. *International Journal of Educational Technology in Higher Education*, *21*(1), 10. https://doi.org/10.1186/s41239-024-00444-7
